# Severe Anemia with Hemoperitoneum as a First Presentation for Multinodular Hepatocellular Carcinoma: A Rare Event in Western Countries

**DOI:** 10.1155/2016/7082387

**Published:** 2016-11-23

**Authors:** Thein Swe, Akari Thein Naing, Aama Baqui, Ratesh Khillan

**Affiliations:** ^1^Department of Internal Medicine, Interfaith Medical Center, Brooklyn, NY, USA; ^2^Department of Pathology, Interfaith Medical Center, Brooklyn, NY, USA; ^3^Division of Oncology, Interfaith Medical Center, Brooklyn, NY, USA

## Abstract

Hemoperitoneum due to spontaneous rupture of hepatocellular carcinoma is a life-threatening and rare condition in western countries with an incidence of less than 3% because of early detection of cirrhosis and neoplasm. Here, we describe a case of a 66-year-old male patient with altered mental status with hemorrhagic shock. Computed tomography scan of abdomen revealed hemoperitoneum and mass in liver. Patient underwent resection of liver tumor and biopsy revealed multinodular hepatocellular carcinoma. A high degree of suspicion is required where severe anemia and hemoperitoneum can be a first presentation for hepatocellular carcinoma especially in patients with chronic hepatitis C infection. Early diagnosis is crucial since mortality rates remain high for untreated cases.

## 1. Introduction

Hepatocellular carcinoma (HCC) is a hypervascular tumor with a high tendency for vascular invasion and can produce growth factors that induce neoangiogenisis. It is one of the most common types of cancer in the world accounting for about 500,000 of new cases diagnosed yearly [1, 2].

Spontaneous rupture of HCC was previously considered as problems of large tumor; however, small tumors with aggressive behavior are also at high risk. Symptoms may vary depending on location of tumor. Although rupture of deep tumors can present with asymptomatic or pain, a peripheral tumor can lead to hemoperitoneum with hemorrhagic shock in severe cases [3]. In the western countries, ruptured HCC is a rare condition with an incidence of less than 3% of HCC patients because of the earlier detection of HCC [2].

## 2. Case Presentation

A 66-year-old male with past medical history of chronic alcoholic and chronic hepatitis C (not sure if it was treated or not) was brought in by Emergency Medical Service (EMS) because of drowsiness and fatigue for 1 day. He denied any history of trauma.

Vital signs were pulse rate of 123 beats/minute, respiratory rate of 23 breaths/minute, and blood pressure of 82/55 mmHg. Physical examination revealed drowsy and pale patient that responded only to painful stimuli. Abdomen examination showed distended, tense abdomen with hypoactive bowel sounds. No organomegaly was found on palpation. Cardiovascular and chest examinations were within normal limits. No external bleeding source was identified.

laboratory tests showed white blood cells of 17.4 × 10^9^/L, hemoglobin of 5 g/dL, hematocrit of 15%, platelet counts of 120,000/*μ*L, mean corpuscular volume of 90.2 fL, absolute reticulocyte count of 63 K./*μ*L (normal = 24–84 K./*μ*L), haptoglobin of 68 mg/dL, bilirubin of 1.5 mg/dL, aspartate transaminase (AST) of 50 IU/L, alanine transaminase (ALT) of 103 IU/L and alkaline phosphatase of 90 IU/L, ammonia of 34 *μ*mol/L, and tumor marker alpha fetoprotein level of 2556 ng/mL (normal = 0–8.3). Serum electrolytes, coagulation profile, amylase, and lipase were within normal limits. Baseline hemoglobin was 14.7 g/dL and hematocrit was 44% in 2011. Occult blood was negative.

**Figure 1:**
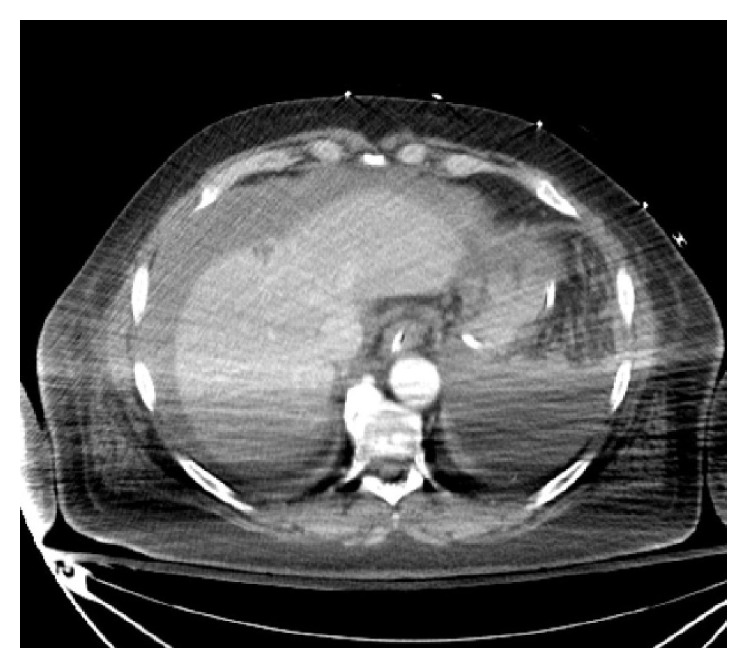
CT scan of abdomen and pelvic revealing hemoperitoneum and mass-like lesion in segment 4 of liver.

**Figure 2:**
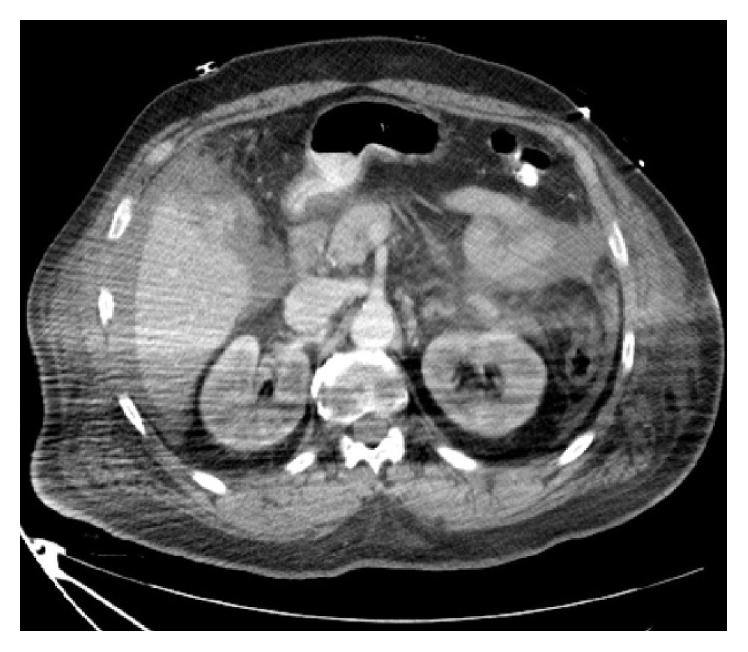
CT scan of abdomen and pelvic showing hemoperitoneum and mass-like density abutting gallbladder.

**Figure 3:**
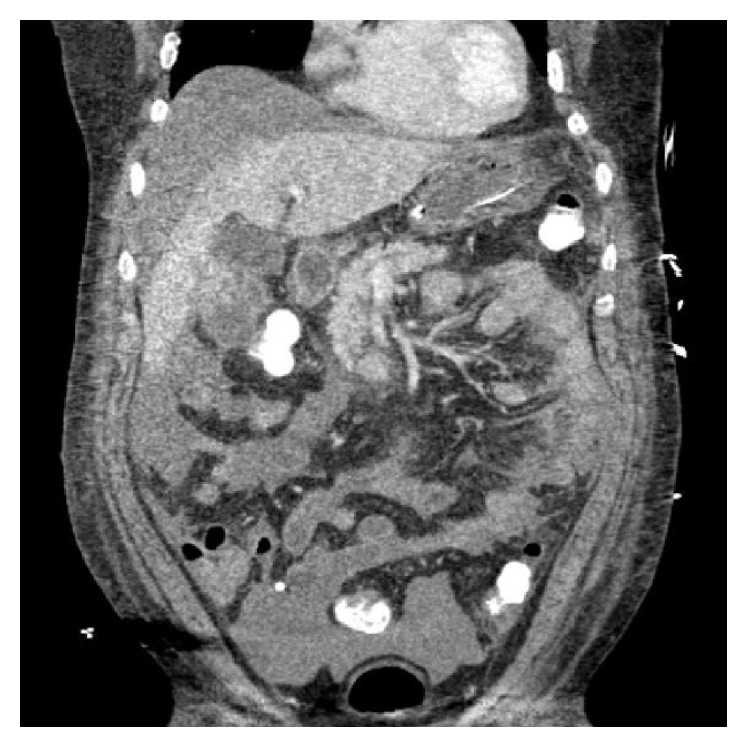
CT scan of abdomen and pelvic showing a mass at segment 5 of liver.

Chest X-ray was unremarkable. Abdominal ultrasound revealed free peritoneal fluid and computed tomography (CT) scan of head showed no acute hemorrhage or infarct. CT scan of abdomen and pelvic revealed hemoperitoneum in the abdomen and pelvis and heterogeneous mass-like density abutting the gallbladder and lower right hepatic lobe (Figures 1, 2, and 3).

## 3. Treatment, Outcome, and Follow-Up

Patient was admitted to the intensive care unit (ICU) and treated with 4-litre bolus of normal saline intravenously and transfused a total of 4 units of pack red blood cells (PRBC). Patient underwent bland transcatheter arterial embolization (TAE). Microcatheter was used along with polyvinyl alcohol (PVA) particles injected into the hepatic artery. A sliding CT scanner system with interventional radiology features (IVR-CT) was used to take CT images during arterial infusion of contrast agents (CT during angiography). A mass was found to be exophytic from the edge of segment 5 of liver adjacent to the gallbladder bed. A small lesion in segment 4A of the liver was also identified. Laparoscopic resections of segments 5 and 4A along with mass and cholecystectomy were performed.

**Figure 4:**
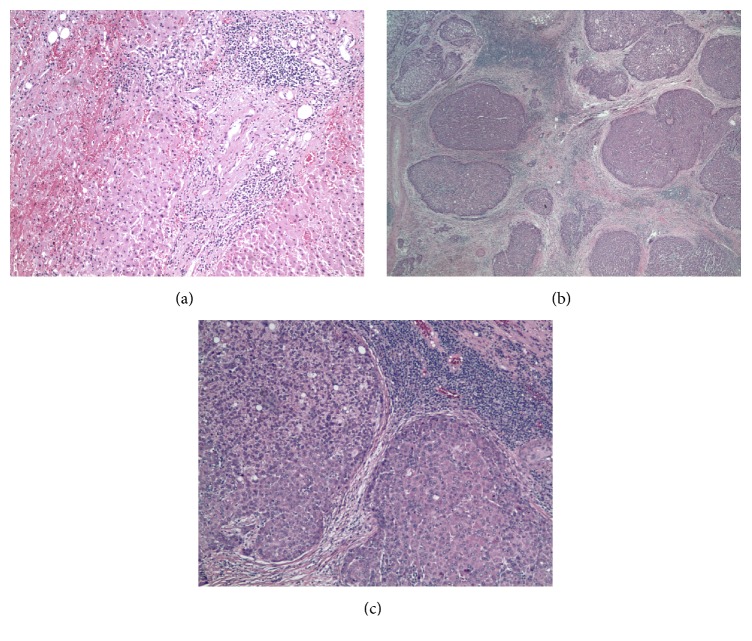
(a) H & E section (40x) shows the background normal liver cells showing mild to moderate portal triaditis and interface hepatitis (grade II) with most portal area infiltrated by many lymphocytes and few plasma cells as typical for hepatitis C. There is mild to moderate septal fibrosis that appears to be periportal and portal-portal (stage II). (b) H & E section (10x) shows multinodular hepatocellular carcinoma with fibrosis around the individual nodules. (c) H & E section (40x) shows poorly differentiated nature of the hepatocellular carcinoma with surrounding significant lymphocytic reaction.

Pathology report revealed that a tumor is segment 5 poorly differentiated 5.3 cm multinodular hepatocellular carcinoma with extensive microvascular invasions. It had approximately 85% necrosis and fibrosis. The tumor in segment 4A was 1.6 cm moderately differentiated multinodular hepatocellular carcinoma with extensive microvascular invasion. Background liver had portal and periportal fibrosis and focal fibrous septum formation. Hematoxylin and eosin (H & E) stained slides revealed evidence of background hepatitis C and grade 2 and stage 2 and multinodular poorly differentiated hepatocellular carcinoma, which was ruptured as evidenced by surrounding blood (Figures 4(a), 4(b), and 4(c)). Some of the hepatocellular carcinoma nodules showed area of steatosis (fatty change). No lymph node was submitted for examination. According to American Joint Committee on Cancer (AJCC), TNM Classification of Malignant Tumours (TNM) stage was T4NxMx, with direct invasion of adjacent organs other than the gallbladder or perforation of visceral peritoneum.

Patient condition improved along with stable vital signs. He tolerated diet gradually and was discharged from the hospital. Patient was called one week later after discharge and he denied any symptoms of postembolization syndrome such as nausea, pain, or fever. He was recommended follow-up with hematology and oncology clinic but was lost to follow-up.

## 4. Discussion

Spontaneous rupture of HCC is more prevalent in male with average age of around 45–75 years [3]. It is a life-threatening condition with a mortality rate at around 25%–75% [3, 4]. The mortality is even higher than that due to bleeding secondary to rupture of oesophageal varices [4]. About half of the cases of HCC occur in cirrhosis due to alcohol abuse while the remaining can be due to chronic hepatitis C and hepatitis B infection since the incidence varies with geographic area and country's socioeconomic status [3].

The symptoms can range from severe abdominal pain to anemia, hemorrhagic shock, and eventually death if left untreated. Kanematsu et al. published a study that states that increased tumor size and extent of extrahepatic protrusion are correlated with an increased risk of rupture of HCC [5]. However, a study by Bassi et al. reported that size of the tumor did not correlate with severity of the hemoperitoneum [3]. It is assumed that a tear in the tumor surface or rupture of a feeding artery is the main reason for rupture of HCC and its complications such as hemoperitoneum [5]. The hepatic artery supplied small tumors and it was drained by the portal vein. Thus, a tamponade effect was created if there is obstruction of the main branches of the portal vein and portal hypertension which eventually lead to tumor rupture [6]. When tumors have an encompassing fibrous capsule which grow expansively, intratumoral pressure can be elevated leading to rupture. CT findings in ruptured HCC through hepatic capsule include discontinuity or disruption of hepatic surface abutting a HCC [5–7].

Management is dependent on hemodynamic status and resuscitation remains the first step in patients with shock. TAE is the first choice of treatment in unstable patient with active intra-abdominal hemorrhage with a success rate of 90% [6]. It improves hemostasis and outcomes. One of the indications for elective surgical treatment is rupture of HCC at an early phase in the development of liver fibrosis because patients with rupture in the terminal phase of liver cirrhosis can be treated conservatively [4]. Conservative management can also be applied to stable patients at initial presentation. However, staged liver resection after securing hemostasis remains a definitive treatment [8]. Although transcatheter arterial chemoembolization (TACE) is recommended as the first line therapy for unresectable hepatic carcinoma, one of rare and serious complications of TACE is rupture of HCC. The predisposing factors are location of the tumor adjacent to the liver capsule, large tumor size, and total occlusion of the feeding artery [9].

Complications include repeated spontaneous rupture and intraabdominal HCC dissemination; however, it is not a contraindication for resection of primary tumor [6]. Prognostic factors to predict survival in the acute phase are serum bilirubin level, hemodynamic status on hospital admission, and prerupture disease state [8]. Tumor rupture itself is an impact on long term survival; however, individuals without portal venous thrombosis and decompensated liver cirrhosis and who underwent curative management had favorable outcome in a long term [10].

The incidence of ruptured HCC is a rare phenomenon accounting for <3% of HCC patients in western countries while incidence is higher in Asian countries in 2.3–26% of all HCC cases [11–15]. The reduced incidence in western countries is thought to be due to early detection of HCC with screening tests and low incidence of viral hepatitis B and hepatitis C. However, about 20–33% of the diagnosis of ruptured tumor is made only during an emergency exploratory laparotomy [16].

It is important to distinguish blood from simple fluid depending on amount of Hounsfield unit (HU) in CT scan images. The attenuation of fluids that have similar density as water such as bile, urine, and intestinal contents ranges from 0 to 15 HU [17]. However, blood usually has higher attenuation than other body fluids and unclotted extravascular blood usually has a measured attenuation of 30–45 HU whereas clotted blood is 45–70 HU [17].

In conclusion, spontaneous rupture of HCC is a rare condition in western countries because of early detection of cirrhosis and neoplasm. However, a high degree of suspicion is required where severe anemia and hemoperitoneum without history of trauma can be a first presenting sign for HCC. Early diagnosis is crucial since mortality rates remain high for untreated cases.
